# Lipoprotein(a), a Lethal Player in Calcific Aortic Valve Disease

**DOI:** 10.3389/fcell.2022.812368

**Published:** 2022-01-27

**Authors:** Jiahui Hu, Hao Lei, Leiling Liu, Danyan Xu

**Affiliations:** ^1^ Department of Cardiovascular Medicine, The Second Xiangya Hospital, Central South University, Changsha, China; ^2^ Research Institute of Blood Lipid and Atherosclerosis, Central South University, Changsha, China; ^3^ Modern Cardiovascular Disease Clinical Technology Research Center of Hunan Province, Changsha, China; ^4^ Cardiovascular Disease Research Center of Hunan Province, Changsha, China

**Keywords:** calcific aortic valve disease, lipoprotein (a), apolipoprotein, oxidized phospholipid, autotaxin

## Abstract

Calcified aortic valve disease (CAVD) is the most common valvular cardiovascular disease with increasing incidence and mortality. The primary treatment for CAVD is surgical or transcatheter aortic valve replacement and there remains a lack of effective drug treatment. Recently, lipoprotein (a) (Lp(a)) has been considered to play a crucial role in CAVD pathophysiology. Multiple studies have shown that Lp(a) represents an independent risk factor for CAVD. Moreover, Lp(a) mediates the occurrence and development of CAVD by affecting aortic valve endothelial dysfunction, indirectly promoting foam cell formation through oxidized phospholipids (OxPL), inflammation, oxidative stress, and directly promotes valve calcification. However, there is a lack of clinical trials with Lp(a) reduction as a primary endpoint. This review aims to explore the relationship and mechanism between Lp(a) and CAVD, and focuses on the current drugs that can be used as potential therapeutic targets for CAVD.

## 1 Introduction

Calcified aortic valve disease (CAVD) is a disease that includes early aortic valve sclerosis and advanced aortic valve stenosis (AVS), characterized by valvular thickening, fibrosis and microcalcification (Osnabrugge et al., 2013; Mathieu and Boulanger, 2014; Lindman et al., 2016). In the United States, there were approximately 2 million patients with CAVD in 2000, and this number is expected to reach 4.5 million by 2030. In addition, there are about 15,000 people who die each year from CAVD (Yutzey et al., 2014). The main treatment for CAVD is surgery or transcatheter aortic valve (AV) replacement. And there are approximately 100,000 patients in the United States who receive this surgery each year. (Go et al., 2014). Currently, there is a lack of effective drug treatment options and an extremely serious disease burden. Therefore, clarifying CAVD pathogenesis is of substantial significance for studying the targets of CAVD intervention and alleviating the social burden.

There are similar risk factors between CAVD and atherosclerosis, including age, smoking, hypertension and hyperlipidemia (Thanassoulis et al., 2010). In the early stage of CAVD, there are also many similarities between the pathological changes to the valvular tissue in atherosclerotic lesions (e.g., lipid retention infiltration, oxidative inflammation, and fibrous calcification reconstruction); but there are many differences between the two diseases with regards to the pathophysiological progress. First, histopathological analysis demonstrated that CAVD exhibited obvious calcification in the early stage (Otto et al., 1994) and was independent of the atherosclerotic process (Cowell et al., 2005; Rossebø et al., 2008; Chan et al., 2010; Demer and Tintut, 2011). Second, the results of multiple independent randomized controlled clinical trials of moderate and severe AVS patients showed that the clinical progress and prognosis of CAVD were not significantly improved after LDL-C lowering treatment with statins alone or statins combined with fibrates (Cowell et al., 2005; Chan et al., 2010; Lindman et al., 2016). In addition, only approximately 40% of CAVD patients undergoing aortic valve replacement simultaneously suffer from severe coronary disease (Kvidal et al., 2000). Therefore, researchers began to explore mechanisms of CAVD other than LDL-C.

Four large-scale randomized controlled clinical trials (ASTRONOMER (Capoulade et al., 2015a), TASS (Moura et al., 2007), SEAS (Rossebø et al., 2008), and SALTIRE (Cowell et al., 2005)) enrolled a total of 2,388 AVS patients aged 58–68 who were treated with rosuvastatin, atorvastatin and simvastatin/ezetimibe. The results showed that the progression of AVS was not significantly alleviated, despite a significant decrease in LDL-C (Teo et al., 2011). These two contradictory results may be related to the simultaneous increase in lipoprotein (a) (Lp(a)), levels induced by statins (Deshmukh et al., 2012; Yeang et al., 2016; Tsimikas et al., 2019). In the ASTRONOMER study, Lp(a) levels increased by 20% 1 year of rosuvastatin treatment (Capoulade et al., 2015a). Similarly, in patients with hyperlipidemia, the levels of Lp(a) increased by 23% in patients with hyperlipidemia after simvastatin/ezetimibe treatment (Yeang et al., 2016). Many other similar studies have shown that the benefits of statin-mediated decreased in LDL-C for AVS may be offset by increased levels of Lp(a) (Insert Table 1 here). Therefore, researchers began to investigate the relationship between Lp(a) and CAVD.

**TABLE 1:** Characteristics of trials which statins significantly increase plasma Lp(a) levels.

Study	Year	Number of patients (placebo/statin)		Statin (dose)	Time (weeks)	Median Lp(a) levels (IQR)in the statin groups	
						Baseline	Follow-up
**Statin VS Placebo Trials**							
MIRACL, Tsimikas et al. (2004a)	2004	2,237 (1,188/1,149)		Atorvastatin (80 mg)	16	10.3 (4.9–28.2)	11.3 (5.0–33.3)
Children with FH, Rodenburg et al. (2006)	2006	177 (86/91)		Pravastatin (40 mg)	104	12.7 (6.1–29.4)	15.1 (6.2–35.2
ASTRONOMER, Capoulade et al. (2015a)	2015	194 (97/97)		Rosuvastatin (40 mg)	52	29.9 (14.1–81.3)	35.0 (18.3–90.7)
**Statin VS Statin Trials**							
O'Donoghue et al. (2014)	2014	2,270 (0/2,270)	1,150	Pravastatin (40 mg)	4	8.9 (3.1–27.5)	9.5 (3.4–42.3)
			1,115	Atorvastatin (80 mg)		6.1 (2.8–22.3)	6.9 (2.6–30.4)
Choi et al., (2008)	2008	250 (0/250)	111	Pravastatin (40 mg)	78	4.2 (2.1–23.5)	4.4 (2.1–28.6)
			139	Atorvastatin (80 mg)		4.2 (2.1–20.6)	4.6 (2.1–40.8)
Yoshida et al., (2013)	2013	42 (0/42)	21	Atorvastatin (10 mg)	12	14.4 (5.1–23.3)	11.3 (5.0–26.2)
			21	Pitavastatin (2 mg)		10.6 (4.0–33.4)	6.7 (4.2–32.1)

Lp(a), lipoprotein(a). FH, familial hypercholesterolaemia; IQR, interquartile range.

There is indeed an increase in Lp(a) levels in patients with CAVD. Ozkan et al., (2019) found that the Lp(a) level in CAVD was 68.67 ± 1.5 mg/dl, and 27.05 ± 1.19 mg/dl in the control group. But the prevalence of elevated Lp(a) in patients with established CAVD is unclear now.

The study by O'Brien et al., (1996) found that lipids in the early calcified valve, and subsequent co-localization results suggested that these lipids may be derived from Lp(a). Lp(a) is a low-density-lipoprotein-like particle that is covalently bound to an apolipoprotein(a) (apo(a)) tail (Romagnuolo et al., 2018). Lp(a) can be deposited in the arterial intima to promote inflammation and foam cell formation, leading to atherosclerosis. Moreover, multiple epidemiological and clinical studies have revealed that Lp(a) is a risk factor for CAVD (Table 2), and high plasma levels of Lp(a) are associated with the rapid progression of CAVD. Therefore, this article will summarize how Lp(a) function as a risk factor for CAVD by mediating its occurrence and development.

**TABLE 2:** Epidemiologic and genetic associations implicating Lp(a) and LPA variants with CAVD.

Author	Year	Study design	Number of patients	Results
Epidemiologic associations				
Gotoh et al. (1995)	1995	Cross-sectional	784 (n = 160 with aortic sclerosis)	36.1% aortic sclerosis in Lp(a) ≥30 mg/dl versus 12.7% in Lp(a) < 30 mg/dl
Stewart et al. (1997)	1997	Cross-sectional	5,201 (n = 1,405 with sclerosis/stenosis)	OR 1.23 (95% CI 1.14–1.32) for top Lp(a) quartile versus lowest
Glader et al. (2003)	2003	Case-control	202 (n = 101 with AVS)	OR 1.7 (95% CI 0.8–2.9) for Lp(a) > 30 mg/dl and 3.4 (95% CI 1.1–11.2) for Lp(a) > 48 mg/dl
Bozbas et al. (2007)	2007	Case-control	285 (n = 112 with AVC)	Lp(a) 27.4 mg/dl in cases versus 19.9 mg/dl in controls
Capoulade et al. (2015a)	2015	Cohort	220 (with mild to moderate AVS) followed for 3.5 ± 1.2 years	Lp(a) > 58.5 mg/dl was associated with 2.6-fold (95% CI 1.4–5.0; *p* = 0.003) increase in odds of rapid AVS progression
Genetic associations				
Thanassoulis et al. (2013)	2013	GWAS (AVC) and prospective cohort (AVS)	CHARGE: 6,942 (n = 2,245 with AVC)	For AVC, OR per G allele = 2.05 (95% CI 1.66–2.53) for rs10455872 in *LPA* gene
			MDCS: 28,193 (n = 308 with AVS)	For AVS, HR per allele in MDCS, 1.68 (95% CI 1.32–2.15) and HR per allele 1.54 (95% CI 1.05–2.27) in CCHS
			CCHS: 10,400 (n = 192 with AVS)	
Kamstrup et al. (2014)	2014	Prospective cohort (AVS)	77,680 (n = 454 with AVS) combined CCHS and CGHS	HR 1.6 (95% CI 1.2–2.1) for a 10-fold genetic Lp(a) increase
Arsenault et al. (2014)	2014	Prospective cohort and case-control replication (AVS)	17,553 (n = 118 with AVS) in EPIC-Norfolk	In incident analysis, HR = 1.78 [1.11–2.87] and HR = 4.83 [1.77–13.20], respectively, for one or two copies of the rs10455872G allele; in case-control, OR 1.57 (95% CI 1.10–2.26)

GWAS, genome-wide association study; AVC, aortic valve calcium; AVS, aortic stenosis.

## 2 Introduction of Lp(a)

Lp(a) is a low-density lipoprotein-like particle synthesized by hepatocytes, which contains a single apoB100 molecule and is covalently linked to a single fibrinogen-like apo(a) molecule through a disulfide bond. Plasma Lp(a) levels are depend on the size of apo(a).

Apo(a) is the unique structure of Lp(a). The evolution of animal apo(a) began with hedgehogs in Europe. Different from humans, the structure of Lp(a) in hedgehog only contains prothrombin-like kringle ring structure Ⅲ (KⅢ). What’s more, apo(a) only present in human, Old World monkeys and orangutans. The structure of apo(a) in human contains a highly variable kringle ring structure Ⅴ (KⅤ). In addition, New World monkeys do not contain apo(a). However, the difference between the Old and New World monkeys was about 6 million years ago. Therefore, the evolution of Lp(a) in human may be a very recent evolutionary event.

The physiologic role of Lp(a) lipoprotein is currently unclear. Some scientists proposed that the potential physiological role of Lp(a) may be to bind and detoxify pro-inflammatory oxidized phospholipids (Hobbs and White, 1999; Tsimikas et al., 2003, 2004b). Since Lp(a) is only present in humans and primates, it may have evolved to provide protection against various oxidative stress. For example, Lp(a) has been proven to participate in wound healing (Yano et al., 1997) and may be involved in preventing angiogenesis in tumor (Trieu and Uckun, 1999).

**FIGURE 1:**
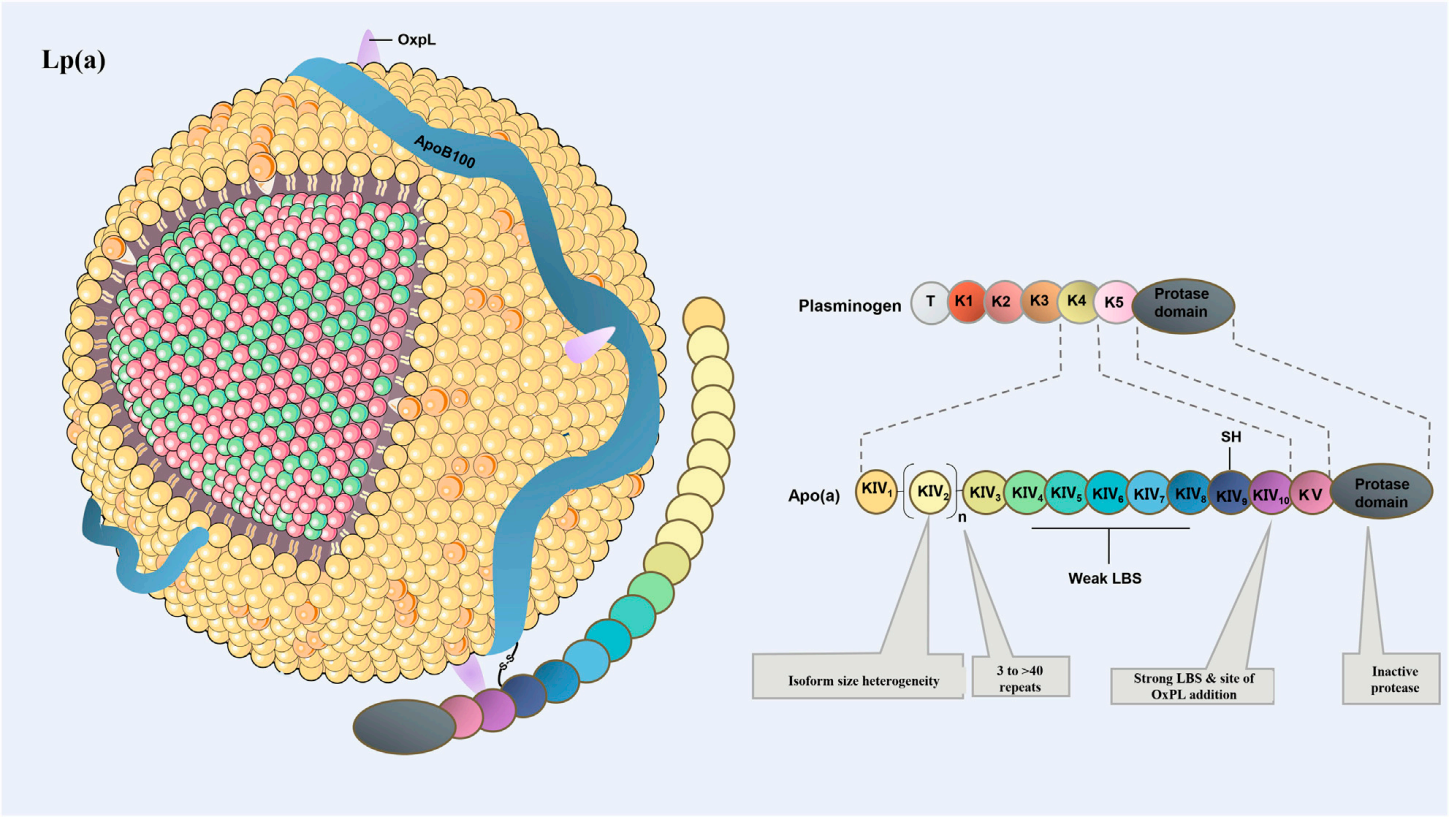
Schematic drawing of the structure of Lp(a). Lp(a) consists of LDL-like microparticles, a single apoB100 molecule, and apo(a). Apo(a) is composed of inactive protease P domain and cyclic domain. Heterogeneity in the number of KⅣ type 2 repeats accounts for different apo(a) isoforms. Except KⅣ_2_, which has multiple copies, the remaining domains are single copy. KⅤ, kringle ring structure Ⅴ; KⅣ, kringle ring structure IV.

In human, Apo(a) consists of one inactive protease-like domain, one copy of kringle ring structure Ⅴ (KⅤ) and multiple copies of KⅣ. There are total of 10 subtypes of KⅣ (KⅣ_1–10_), of which the variable number of KⅣ_2_ modules provide the basis for the size and heterogeneity of apo(a) isomers (Clarke et al., 2009; Kamstrup et al., 2014; Boffa and Koschinsky, 2019; Gudbjartsson et al., 2019) (Insert Figure 1 here). Copies of the *LPA* gene affects the molecular size of apo(a) primarily by influencing the number of KⅣ_2_ copies.

The KV domain assists apo(a) in inhibiting the plasminogen activation system on the fibrin surface to some extent. This in turn inhibits the degradation of fibrin and increases the risk of thrombosis, and has the potential for MI and ischemic stroke in the plaque rupture (Hancock et al., 2003; Wilson et al., 2019). Lp(a) can also bind to macrophages with a high affinity, promoting the formation of foam cell and lipid plaques, which accelerats cholesterol deposition in atherosclerotic plaques (Reyes-Soffer et al., 2017). More importantly, Lp(a) can transport oxidized phospholipids (OxPL) in the plasma through covalent binding (Tsimikas et al., 2005; Boffa and Koschinsky, 2019). In human body, approximately 85% of OxPL is carried by Lp(a), OxPLs, might hold the key to Lp(a) pathogenicity and provide a mechanistic link between Lp(a) and CAVD. OxPLs are predominantly covalently bonded to apo(a) but are also found covalently linked to apoB100 and free in the lipid moiety (Boffa and Koschinsky, 2019). What’s more, there was a strong correlation between levels of Lp(a) lipoprotein and the OxPLs: apo B-100 ratio (r = 0.83, *p* < 0.001) (Tsimikas et al., 2005). Therefore, OxPL is a key factor of ASCVD and CAVD caused by Lp(a).

## 3 Epidemiologic and Genetic Associations Between Lp(a) and CAVD

The plasma Lp(a) concentration has been correlated with CAVD. AVS is the most common heart valve disease, affecting nearly 3% of the population over 65 years old in the West (Otto, 2008). Adverse cardiovascular events include heart failure, valve replacement, or death within 2–5 years of follow-up (Novaro et al., 2007). It is estimated to affect 2–7% of the pop-prevent aortic valve disease progression exists, and aortic valve replacement, costly and associated with high perioperative mortality, remains the only treatment option (Iung and Vahanian, 2011; Thanassoulis et al., 2013; Vahanian et al., 2021). Therefore, it is hoped to identify the causal risk factors in order to provide an opportunity for prevention. Cross-sectional studies have shown that patients with AVS detected by clinical diagnosis or ultrasound exhibit higher levels of Lp(a) (Gotoh et al., 1995; Stewart et al., 1997). A recent observational study also found that increased Lp(a) is an independent predictor of AVS occurrence, rapid progression, and AVS-related events (e.g., aortic replacement and death) in mild to moderate AVS patients (Capoulade et al., 2015a). In asymptomatic, heterozygous patients with familial hypercholesterolemia treated with statins, the probability of AVS increased by 11% (95% CI 1.01–1.20) for every 10 mg/dl increase in Lp(a) (Vongpromek et al., 2015). In the European Cancer Prospective Survey-Norfolk study, which followed 17,533 patients were followed for an average of 11.7 years. The results revealed that patients with Lp(a) ≥ 50 mg/dl had a 1.98-fold (95%CI: 1.25–3.09) increased risk of developing AVS after adjusting for confounding factors, such as age, sex, smoking, and LDL-C (Arsenault et al., 2014).

80% of the population’s plasma Lp(a) level is 0 mg/dl–50 mg/dL^30^. A secondary analysis of the ASTRONOMER trial, followed up 220 patients with mild to moderate aortic stenosis, found that the probability of AVS increased by 11% for every 10 mg/dl increase in Lp(a) (95%CI 1.01–1.20) (Vongpromek et al., 2015). Moreover, the study by Kamstrup et al., (2014) integrated two large-scale prospective studies, the Copenhagen City Heart Study (n = 10,803) and the Copenhagen General Population Study (n = 66,877), found that: the risk of AVS increased by 20% when the Lp(a) level was 5 mg/dl–19 mg/dl; the risk of AVS increased by 60% when the Lp(a) level was 20 mg/dl–64 mg/dl; the risk of AVS increased by 100% when the Lp(a) level was 65 mg/dl–90 mg/dl; and the risk of AVS increased by 2.9 times when Lp(a) > 90 mg/dl, confirming that there is a strong dose-dependent increased risk of AVS in those with elevated Lp(a) levels.

At present, several studies have confirmed that the elevated Lp(a) level indicates increased risk of AV replacement and faster hemodynamic progress in AS (Vongpromek et al., 2015; Ljungberg et al., 2017; Guddeti et al., 2020). The ASTRONOMER study has shown that patients with the highest quartile of Lp (a) levels had higher incidence of clinical events, including AVR and cardiac death (Vongpromek et al., 2015). Recently, Pérez de Isla et al., (2021) also found that Lp(a) is a predictor of aortic valve replacement in familial hypercholesterolemia.

More importantly, in a retrospectively analysis, Ma et al., (2019) found that, patients with Lp(a) > 30 mg/dl had higher incidence of paravalvular leak and the greater historical rates of coronary heart disease requiring PCI/CABG after following transcatheter aortic valve replacement (TAVR). However, there was no association in the incidence of periprocedural complete heart block requiring permanent pacemaker placement following TAVR, major bleeding, necessity of conversion to surgical valve replacement, 30-days mortality of CV, nonfatal MI, or postprocedural CVA (Ma et al., 2019). And further research in larger cohorts is needed to confirm the prevalence of elevated Lp(a) levels in structural deterioration of prosthetic valves.

Although several studies support the association between Lp(a) and CAVD (Insert Table 2 here), these observational studies cannot confirm whether Lp(a) is a risk factor for CAVD or just a simple marker of the disease. This question concerns whether Lp(a) can become a therapeutic target for CAVD. Therefore, some subsequent genetic studies have provided an important basis for the causal relationship between Lp(a) and CAVD.

Genetic studies have found that *LPA*, the gene encoding Lp(a), is related to CAVD. Genome-wide association study (GWAS) and cohort study have found that single nucleotide polymorphisms (SNPs) at the *LPA* gene locus are correlated with the occurrence of CAVD (Thanassoulis et al., 2013; Arsenault et al., 2014). The Heart and Aging Research Group of the European Genome Epidemiology Consortium (CHARGE Consortium) found that the rs10455872SNP in the *LPA* was the only single nucleotide polymorphism associated with aortic valve calcification in the whole genome of European Caucasians, African Americans, and Latino Americans through computer tomography (CT) scans, which reached genome-wide significance for the existence of aortic valve calcification. Therefore, rs104558722SNP could double the increase the incidence of aortic valve calcification (Thanassoulis et al., 2013). Moreover, a Mendelian randomization analysis showed that genetically determined Lp(a) levels were causally associated with a 62% increase in the odds of aortic-valve calcification per log-unit increment in the level of Lp(a) (Thanassoulis et al., 2013). It is suggested that the risk of CAVD caused by rs10455872SNP is closely related to the level of Lp(a).

Clarke et al., (2009) confirmed that rs10455872 gene mutations reduces the repeats of the KⅣ_2_ loop domain by affecting the copies of *LPA*, resulting in increased level of Lp(a). In addition, the Mendelian randomized study in Copenhagen also showed that when the percentage of KⅣ_2_ repeat sequences were >79%, 35%–79%, 12%–34%, 6%–11%, and <6%, respectively, the Lp(a) levels were 8 mg/dl, 12 mg/dl, 23 mg/dl, 49 mg/dl, and 65 mg/dl. (Kamstrup et al., 2014). It is suggested that the level of Lp(a) increases with a decrease of KIV_2_ repetitions in *LPA*. More importantly, another Mendelian randomized study in Copenhagen showed that in patients with rs10455872 non-carriers, rs10455872 heterozygotes and homozygotes, the median Lp(a) levels were 11 mg/dl, 60 mg/dl, and 108 mg/dl, and the risk ratios of CAVD in heterozygotes and homozygotes were 1.6 and 1.59, respectively (Kamstrup et al., 2014). Not only did the genetic level confirmed that there was a strong hierarchical relationship between the elevated levels of Lp(a) and the occurrence of CAVD, but more importantly, it is also confirmed that the genetic variations in *LPA* can increase the risk of CAVD by affecting the levels of Lp(a).

In addition to rs10455872SNP, rs3798220SNP may also be related to CAVD; however, current studies have shown that this gene has a lower frequency of minor allele mutations and has little effect on the levels of Lp(a), which does not reach the genome-wide significance (Table2).

## 4 Lp(a) Participates in the Pathophysiological Process of CAVD

For many years, the mechanism of CAVD has been recognized as a type of aortic valve tissue degeneration. The valve is worn under the irreparable mechanical damage caused by hemodynamics, and the deposition of calcium and phosphate in valve tissue irreparably. However, the current cognition of the mechanism of CAVD has been transformed into a “highly regulated autonomous process” by the traditional concept of “degenerative of calcium and phosphorus deposition".

**FIGURE 2:**
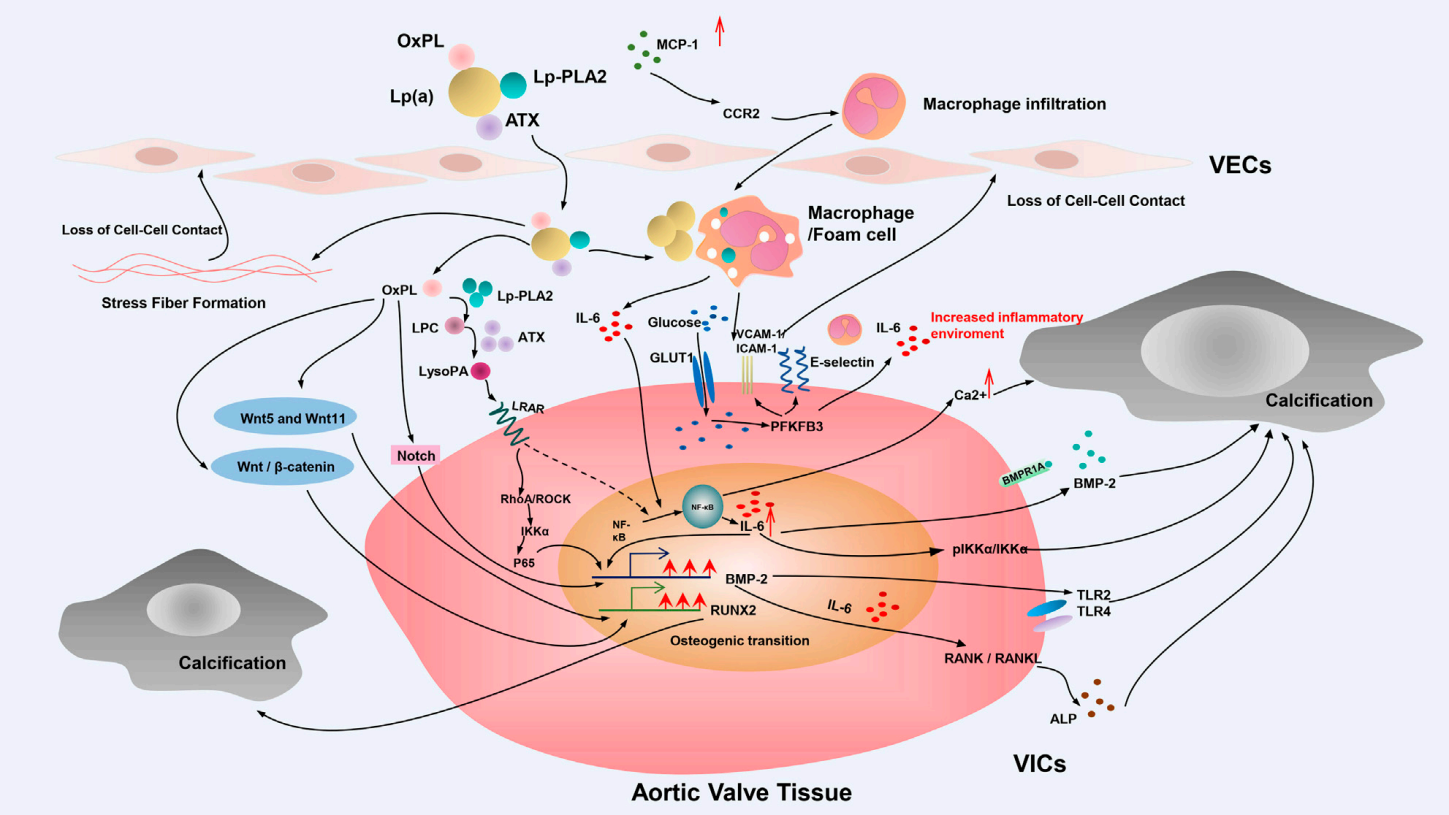
Mechanisms of Lp(a)-induced CAVD. Schematic diagram depicts the mechanism of Lp(a)-induced CAVD. The main pathophysiological processes involving Lp(a) in CAVD include endothelial dysfunction, indirect promotion of foam cell formation through OxPL, valvular calcification and so on. Valvular calcification of VICs plays an important role in the occurrence and development of Lp(a)-mediated CAVD. This process of phenotypic transformation is mainly including the Notch signal pathway and NF-κB receptor activator pathway, and is also partially mediated by inflammation, oxidative stress, and the Wnt signal pathway. OxPL, oxidized phospholipids; Lp(a), lipoprotein (a); ATX, autotaxin; Lp-PLA2, lipoprotein-associated phospholipase A2; MCP-1, monocyte-macrophage chemoattractant protein-1; CCR2, C-C chemokine receptor type 2; LPC, lysophosphatidylcholine; LysoPA, lysophosphatidic acid; LPAR, lysophospholipid receptor; GLUT1, glucose transporter type 1; VCAM-1, vascular cell adhesion molecule-1; ICAM1-1, intercellular adhesion molecule-1; IL-6, interleukin 6; BMP-2, bone morphogenetic protein-2; IKKα, IkappaB kinase-alpha; TLR,; Toll-like receptor; VECs, endothelial cells; VICs, valvular interstitial cells.

In the pathogenesis of CAVD, the incomplete understanding of the role of Lp(a) at the molecular level and the absence of appropriate animal models are the current difficulties and dilemmas barriers for the development of specific and effective clinical interventions designed to mitigate the role of Lp(a)—mediated CAVD. At present, the main pathophysiological processes involving Lp(a) in CAVD include endothelial dysfunction, indirect promotion of foam cell formation through OxPL, valvular calcification, as well as inflammation, oxidative stress, and the direct promotion of valvular calcification and so on (Figure 2).

### 4.1 Lp(a) Affects the Function of Valve Endothelial Cells in CAVD

Endothelial cell integrity is necessary for aortic valve function to maintain a dynamic balance. Morever, endothelial barrier dysfunction is a key event in the early stage of CAVD, which primarily manifests by increased permeability and changes in endothelial secretion characteristics, resulting in impaired endothelial adhesion function (Tao et al., 2012; Gould et al., 2013).

The key to normal endothelial barrier function is the actuation/myosin drive-mediated endothelial contraction process. The loss of endothelial barrier function is primarily due to the activation of endothelial contractile function (Michel and Curry, 1999), which leads to the opening of adjacent cell gaps followed by changes in endothelial cell permeability.

Multiple studies have confirmed that apo(a) is a pro-inflammatory apolipoprotein. Following activation of Rho, RhoK (the effector protein of Rho), increases myosin light chain (MLC) phosphorylation by increasing myosin phosphatase target subunit 1 (MYPT1) phosphorylation, and decreasing MLC phosphatase activity. This finding promotes the formation of actin/myosin stress fibers (Babaei et al., 2003; Cho et al., 2008; Liu et al., 2013), ultimately resulting in increased ECs permeability ultimately. It is suggested that apo(a) may play a role in increasing the permeability of ECs through Rho/RhoK-dependent signal pathway (Babaei et al., 2003; Cho et al., 2008; Liu et al., 2013).

Based on this theory, Cho et al., (2008) found that when human umbilical vein endothelial cells (HUVECs) were treated with 17K Δ Asp recombinant apo(a) (17KΔAsp r-apo(a)) with a single amino acid to replace of lysine binding site (LBS) in KⅣ_10_, there were no significant changes in MLC phosphorylation, actin stress fibers, or endothelial permeability in HUVECs. In contrast, after intervention with other recombinant apo(a) (17K r-apo(a)) with strong LBS function retention in KⅣ10, such as functional structure deletion mutations (e.g., KIV_1–4_, KIV_5_-P, KIV_5–10_, and KV), increased MLC phosphorylation and formation of F-actin stress fibers with different degrees in HUVECs was observed. These changes were associated with a simultaneous increase in endothelial cell permeability. In HUVECs treated with Rho kinase inhibitor, MLC phosphorylation, formation of F-actin stress fibers, and increased of endothelial cell permeability induced by 17Kr-apo(a) were inhibited. These findings confirmed that the increase in endothelial permeability induced by apo(a)-mediated Rho activation was dependent on the function of LBS in the KⅣ_10_ domain.

Furthermore, this team also found that after treated with lysine analogues, a compound that could eliminate the increased ECs permeability induced by physiological apo(a), the increase in MLC phosphorylation, F-actin stress fiber formation, and endothelial permeability mediated by 17Kr-apo(a) were completely inhibited in a dose-dependent manner, similar to that observed with Rho kinase inhibitors (Cho et al., 2008). This finding suggests that apo(a) may also increase ECs permeability through the lysine pathway expressed on the cell surface.

Endothelial cell barrier function also depends on the normal adhesion function between cells, whereas Lp(a) can induce a loss in intercellular adhesion function by affecting the expression and secretion of various proteins in the endodermis. Schnitzler et al., (2020) found that the level of ICAM-1 expression, a molecule involved in intercellular adhesion, was up-regulated in patients with elevated Lp(a). *In vitro*, human aortic endothelial cells (HAECs) were incubated with normal monocytes after Lp(a) was added, it was found that the expression of ICAM-1 and VCAM-1 increased, suggesting that apo(a) could induce the secretion and affect the adhesion of HAECs.

### 4.2 OxPL Is a Key Factor of Lp(a)-Mediated CAVD

Lp(a) is the main carrier of OxPL. 85% lipoprotein-related OxPL in human plasma is connected to the apo(a) (Leibundgut et al., 2013), and plays a pathogenic role. On the one hand, OxPL is able to directly attract circulating monocytes and promote their transformation into macrophages. Macrophages can phagocytose oxidized lipids, becoming foam cells that can cause disease. On the other hand, the phosphocholine (PC) of OxPL in oxidized LDL (ox-LDL) can be recognized by macrophage scavenger receptors and Toll-like receptors (TLRs), the natural antibody (NAb), which can be considered immunoglobulin pattern recognition receptors (PRRs), and natural IgM antibodies, E061, which promotes inflammation in CAVD (Miller et al., 2011; Binder et al., 2016). In addition, OxPL can also interact with signal transduction receptors and specific binding sites (e.g., CD36, TLR2/1, TLR 6, TLR4, and CD14) on the macrophage cell surface, and simultaneously up-regulate the expression and secretion of various cytokines related to AVS and endothelial cells inflammation (Podrez et al., 2002a; Imai et al., 2008; Kadl et al., 2011; Lee et al., 2012; Popat et al., 2017; Zanoni et al., 2017).

Based on these characteristics, OxPL plays a critical role in CAVD (Capoulade et al., 2018; Zheng et al., 2019) s a key factor for pathogenic effect of Lp(a) (Tsimikas et al., 2005; Tsimikas and Witztum, 2008; Capoulade et al., 2015a). In addition, epidemiological studies have shown that the levels of Lp(a) and OxPL are closely related to the incidence of CAVD (Capoulade et al., 2015a, 2018). In 2015, Capoulade et al., (2015a) conducted a statistical analysis of the ASTRONOMER study and found that patients in the highest quartile for Lp(a) levels or those in the highest quartile for OxPL-apoB levels were associated with a higher incidence of aortic valve replacement, suggesting that increased levels of Lp(a) and OxPL-apoB were associated with accelerated AVS progression. Interestingly, in a subsequent follow-up of 220 patients with mild to moderate AVS and a secondary analysis of the ASTRONOMER trial, they found there was a linear relationship between the rapid progression of calcified aortic stenosis (CAVS) and the proportion of OxPL in Lp(a) (Capoulade et al., 2018), supporting the previous conclusion. Another research with two prospective cohort studies containing 145 patients with AVS over a median follow-up of 5 years, it was found that 59.2% of these patients in the highest quartile for Lp(a) and OxPL had a main combined outcome of aortic valve replacement (AVR) or all-cause mortality, and 34.4% of these in the lowest quartile of Lp(a) and OxPL had the main combined outcome of AVR or all-cause mortality. These findings suggest that patients with high serum Lp(a) and OxPL levels have an increased risk of AVR and all-cause mortality (Zheng et al., 2019). Taken together, these reports confirm that Lp(a) and OxPL can drive AV calcification and accelerate disease progression.

At present, OxPL-induced CAVD is primarily caused by abnormal endothelial cell migration, foam cell formation, and valvular osteogenic calcification (e.g., different signal pathways, pro-inflammatory response, and oxidative stress).

#### 4.2.1 OxPL Promotes Abnormal Endothelial Cell Migratory Function

OxPL plays an important role in the impairment of endothelial cell migration induced by Lp(a). After added 17-kringle recombinant apo(a) (17K r-apo(a)) containing OxPL and recombinant apo(a) without OxPL (17KΔOxPL r-apo(a)) respectively in HAECs, the ability of transendothelial migration and the expression of SELE, ICAM-1 and VCAM-1 were increased in the 17K r-apo(a) group, whereas no significant changes were observed in the 17KΔOxPL r-apo(a) group. In contrast, after adding the mouse IgM monoclonal antibody, E06, to bind to the PC region of OxPL, there was decreased transendothelial migration and the expression of cell adhesion-related molecules of HAECs decreased in the 17K r-apo(a) group. However, these indexes remained unaffected in the 17KΔOxPL r-apo(a) group (Chang et al., 2004; Scipione et al., 2015), suggesting that the endothelial adhesion functionality of ECs mediated by Lp(a) is regulated by the presence of OxPL on Lp(a).

More interestingly, Schnitzler et al., (2020) also found that Lp(a) could upregulate the expression of glycolysis regulator 6-phosphofructose-2-kinase/fructose-2,6-bisphosphatase-3 (PFKFB-3) in CAVD patients. In ECs stimulated with 17K r-apo(a) or Lp(a) for 6 h, there was increased secretion of lactic acid, as well as the expression of HIF1β and KLF2, downstream molecules of PFKFB-3, suggesting that Lp(a) can increase glycolysis and its regulator, PFKFB-3, in ECs. However, when a glycolysis inhibitor was added or PFKFB-3 was knocked down, the expression of glycolysis, PFKFB-3, and the related downstream molecules were substantially decreased. At the same time, the transendothelial migration ability of ECs and the expression of SELE, ICAM-1, and VCAM-1 were decreased. These results indicate that Lp(a) can up-regulate PFKFB-3 expression through OxPL and mediate glycolysis to activate endothelial function, leading to the damage of intercellular adhesion function, and impairing the endothelial function.

#### 4.2.2 OxPL Promotes Foam Cell Formation in CAVD

Lipid accumulation and deposition play an important role in the early occurrence and development of CAVD. In the development of CAVD, similar to atherosclerosis, after the aortic valve endothelium is injured, there is an infiltration and accumulation of lipids in the intima or fibrous layer, and subsequently recruit macrophages. When overloaded with lipids, these macrophages will transform into foam cells, and cause CAVD (Rajamannan, 2009).

The study conducted by Otto et al., (1994) found that there was no lipid pool in the normal intact tissue adjacent to the AV lesions, but there was lipid infiltration in the damaged and calcified AV (O'Brien et al., 1996). This finding suggests that lipid accumulation may be involved in the AVECs damage in the diseased AV Solache-Berrocal et al., (2019) found that a large amount of CD68-positive immunostaining in the calcified aortic valve area in patients undergoing aortic valve replacement, which were subsequently identified as macrophages. While no CD68-positive staining was observed in the non-calcified area, further studies have shown that there is a correlation between the CD68-positive region and the ratio of bone volume/tissue volume (BV/TV). These results indicate that the degree of macrophage infiltration is related to the degree of AV calcification. Previous histological studies have already confirmed that apolipoprotein apoB and apo(a) exist in surgically resected stenotic AV (Capoulade et al., 2015b). Thus, it is speculated that Lp(a) may promote the occurrence and development of CAVD through lipid accumulation in macrophages.

Lp(a) is the main carrier of OxPL (Leibundgut et al., 2013). Importantly, previous animal experiments have confirmed that OxPL containing phosphatidylcholine can cause lipoproteins to enter macrophages via scavenger receptors (i.e., CD36 and SR-A) without regulation by other cell factors (Miller et al., 2011), thereby promoting the formation of foam cells (Podrez et al., 2002b). Moreover, Chen et al., (2018) found that macrophage infiltration and expression of the CCR2 ligand, monocyte-macrophage chemoattractant protein-1 (MCP-1), were increased in the fibrotic aortic valve in aging accelerated mouse model. Kadl et al., (2009) also found that OxPAPC, a mixture of various types of OxPL derived from radical oxidation, could increase the expression of MCP-1. It has previously been clarified that CCR2 plays an important role in monocyte recruitment induced by OxPAPC (Kadl et al., 2009). It can be inferred that under appropriate environmental conditions, oxidizztion through OxPL or various OxPL derivatives, Lp(a) can promote t MCP-1-mediated f CCR2 expression to recruit mononuclear macrophages. Subsequently, these oxidized Lp(a) are transferred into macrophages to mediate foam cell formation through scavenger receptors, CD36 and SR-A, which plays an important role in foam cell formation during the early stages of CAVD (O'Brien et al., 1996). However, few related experiments *in vitro* and *in vivo* experiments have been performed, and the specific mechanism remains to be further studied.

#### 4.2.3 OxPL Promotes Valvular Osteogenic Calcification in CAVD

The aortic valve consists of endothelial cells (VECs) and valvular interstitial cells (VICs) (Jiang et al., 2002; de Lange et al., 2004). The VICs of the mature aortic valve are activated during CAVD formation. The conversion of VICs to an osteoblast-like phenotype is considered to represent a basic step and key factor in the acceleration of valvular calcification (Poggio et al., 2013). This process of phenotypic transformation is mainly driven by calcification regulation pathways, including the Notch signal pathway and NF-κB receptor activator pathway, and is also partially mediated by inflammation, oxidative stress, and the Wnt signal pathway.

##### Notch Signaling Pathway

Notch-1, a member of the Notch family, is a transmembrane receptor in mammals. The specific destruction of Notch1 has shown the lethality of embryos (Swiatek et al., 1994; Krebs et al., 2000). Notch-1 is related to cell fate, outflow tract formation and endocardium-mesenchymal transition, which is crucial for the formation of aortic valve and pulmonary valve (Lee et al., 2019).

Notch-1 plays an important role in the development of the aortic valve during embryogenesis. Studies have confirmed that Notch-1 mutations are related to the occurrence of aortic valve disease (Prakash et al., 2014). Notch-1 can inhibit calcification through inhibiting the osteoblast transcription factor, RUNX2 (Ducy, 2000; Kaden et al., 2004; Garg et al., 2005; Nigam and Srivastava, 2009; Rajamannan et al., 2011). However, Notch-1 also participates in early stages of valve calcification by stimulating another osteoblast transcription factor, bone morphogenetic protein-2 (BMP-2) (Nigam and Srivastava, 2009). Importantly, studies have shown that BMP-2 is a key protein involved in the process of osteoblast-like phenotypic transformation and aortic valve calcification in VICs (Pawade et al., 2015).

*In vitro*, it was found that the osteogenic differentiation induced by Lp(a)-mediated RUNX2 and BMP2 can be attenuated by a pre-incubation of Lp(a) with an E06 monoclonal antibody in ECs (Zheng et al., 2019). In addition, it has been speculated that OxPL represents an important factor in Lp(a)-mediated calcification and osteogenic differentiation. Previous studies have also shown that OxPL is a TLR-2 and TLR-4 ligand (Kadl et al., 2011). Both TLR-2 and TLR-4 are the main promoters of BMP-2 osteogenic differentiation, and are the key factors involved in bone and cartilage formation. Several studies have found that the proportion of OxPL and TLR-4 expression is increased in calcified aortic valves (Caira et al., 2006; Yang et al., 2009). Furthermore, the expression of RUNX2 and BMP2 was up-regulated after added 17Kr-apo(a) in ECs. However, when the covalent binding ability between OxPL and Lp(a) was affected, the expression of RUNX2 and BMP2 genes disappeared (Zheng et al., 2019), confirming that the covalent binding between OxPL and Lp(a) plays an important role in the osteogenic differentiation of endothelial cells. These results suggest that OxPL may participate in osteogenic differentiation in AV calcification through the Notch-1—TLR-2/TLR-4—RUNX2/BMP-2 pathway.

In addition, BMP-2 can be up-regulated by the combination of RANKL and RANK. The activation of the RANK/RANKL pathway can promote CAVD via the formation of calcification-related proteins (e.g., alkaline phosphatase [ALP] and osteocalcin). Pawade et al. (2015); Zheng et al., (2019) also found that a pre-incubation of Lp(a) with the E06 monoclonal antibody could attenuate the osteogenic differentiation of Lp(a)-mediated IL-6 and could further decrease the expression of IL-6, RANKL-11, and valvular calcification after an intervention with 17KΔOxPL r-apo(a). Previous studies have shown that IL-6 can induce the expression of tumor necrosis factor superfamily member 11 (RANKL-11) in osteocytes. (Wada et al., 2006). It is suggested that OxPL may also participate in osteogenic differentiation of AV calcification through the Notch-1—BMP-2—IL-6—RANKL-11 pathway.

##### ATX—NF-κB Receptor Pathway

Autotaxin (ATX) is a lysophospholipase D enzyme that was initially identified as a motility factor in a melanoma cell line (Umezu-Goto et al., 2002). It mainly hydrolyzes lysophosphatidylcholine (LPC) to lysophosphatidic acid (LysoPA) (Stracke et al., 1992). At the transcription level, the expression of ATX is mainly affected by chromatin modification and different transcription factors (e.g. AP-1, NF-κB, nuclear factor of activated T cells (NFAT1), signal transducer and activator of transcription 3 (STAT3)). Some inflammatory cytokines, such as TNF-α, IL-6 and IFNα/β, can also influence the expression of ATX by inducing some different downstream signal cascades. At the post-transcriptional level, RNA binding proteins (HuR and AUF1), RNA methyltransferase (NSUN2) and some microRNAs (e.g. miR-101-3p) can affecting the stability and nuclear output and translation of ATX mRNA. The expression of ATX can be down-regulated through the negative feedback loop of LPA (Zhang et al., 2021). In addition, the secretion of ATX is also a regulated process, dependent on the hydropho-bic core sequence of the signal peptide (Jansen et al., 2005).

Studies have recently noted that ATX is enriched in the Lp(a) lipid fraction and circulating Lp(a) particles associate with ATX and enter the aortic valve. Furthermore, laser confocal analysis has shown that apo(a) immunofluorescence was co-distributed with ATX in calcified AV (Bouchareb et al., 2015), and the activity of ATX increased in calcified aortic valve tissue mediated by Lp(a) and OxPL (Bouchareb et al., 2015). These findings indicate that ATX may play an important role in the pathogenesis of OxPL-induced CAVD.

Lp(a) fragments isolated from calcified AV also showed increased activity of ATX and its enzymes. It is speculated that Lp(a) can mediate AV calcification by affecting the activity of both ATX and its associated enzymes. Salles et al., (2013); Bouchareb et al., (2015) found that the plasma ATX content and activity in patients with CAVD were higher than those in the control group. Further analysis showed that after adjusting for age and sex, the risk of CAVD increased by 3.5 times in patients with high activity of ATX (≥84RFUmin-1, median) and higher Lp(a) level (≥50 mgdL-1, median), compared with patients with lower activity of ATX and Lp(a) levels. In addition, the risk of CAVD increased by 5.5 times in patients with higher ATX activity and OxPL-apoB (≥2.02 nmol/L, median). (Salles et al., 2013). It was further confirmed that Lp(a) and OxPL could mediate CAVD occurrence and development by affecting the ATX activity, as the activity of ATX increases, the risk of CAVD increases.

Based on the above results, many epidemiological studies related to activity of ATX—Lp(a)/OxPL—CAVD prompted scholars to conduct in-depth research into the related mechanisms.

In addition to the increased ATX activity observed in the calcified aortic valves, Bouchareb et al., (2015) also found that the conversion of lysophosphatidylcholine (LPC) to lysophosphatidic acid (LysoPA) was higher in the stenotic aortic valve than that in non-calcified tissues. It has also been confirmed that ATX can metabolize LPC presents in the Lp(a) particles into LysoPA (MacPhee et al., 1999), and LPC can promote osteoblasts differentiation in VICs mediated by OxPL. Mahmut et al. (2014a) LysoPA is the ligand for lysophospholipid receptor (LPAR) (Boutin and Ferry, 2009), which plays an important role in osteogenesis. Importantly, mice lacking LPAR exhibit decreased bone mass and osteogenic ability (Salles et al., 2013), suggesting that the high level of ATX activity in calcified AV may be related to the LPC and LysoPA production. In addition, chromatographic tandem mass spectrometry confirmed the existence of LysoPA in explanted calcified AV. Moreover, the LysoPA content exceeded the level of LPC (Bouchareb et al., 2015), with an odds ratio of 16:0 and 18:1. Torzewski et al. (2017) This finding indicates that the calcification process may be related to the transformation of LPC to LysoPA in valvular leaves promoted by ATX. The hydroxyapatite and calcium deposition was significantly increased in the AV of LDLR^–/–^/apoB_100_^-/-^/IGFII transgenic mice treated with LysoPA for 6 months, and the expression of BMP2 in the aortic valve lobule was also increased, resulting in accelerated progression of aortic valve stenosis phenotype. (Bouchareb et al., 2015). However, it was found that the Ca^2+^ deposition, calcification, and expression of BMP2 in VICs induced by LPC and LysoPA was decreased after blocking LPAR, and the phenotypic progression of aortic stenosis slowed down, (Bouchareb et al., 2015), suggesting that valvular calcification induced by LPC was dependent on LysoPA. Therefore, ATX promotes the occurrence and development of CAVD by inducing the transformation of LPC into LysoPA.

LysoPA is a bioactive lipid metabolite, and the combination of LysoPA and LPARs can promote the nuclear translocation of NF-κB. Bouchareb et al., (2015) found that the level of ATX mRNA in the stenotic aortic valve was positively correlated with the expression of IL-6 mRNA, a known downstream target of NF-κB (Brasier, 2010). In addition, studies have shown that the ratio of phosphorylated IKKα(Ser 176–180)/IKKα (regulated by the NF-κB pathway) was increased in calcified AV (Brasier, 2010). It is speculated that the NF-κB - IL/6 signaling pathway may play an important role in the development of CAVD induced by ATX—LysoPA.

Cho et al., (2008) found that the p65 subunit of NF-κB was gradually transferred from the cytoplasm into the nucleus within 30 min in the VICs exposed to LPC, indicating that the NF-κB pathway was activated. The team has previously confirmed that the activation of the NF-κB pathway in VICs promotes IL-6 production, which stimulates BMP2 in a paracrine manner to mediate valve leaflet calcification. However, whether inhibiting NF-κ B pathway, silencing IL-6 expression or inhibiting BMPs, LPC-induced VIC calcification could be inhibited (Bouchareb et al., 2015), confirming that LPC-mediates VICs calcification through the NF-κB—IL-6—BMP pathway. Moreover, after treated with LysoPA in VICs, both the expression of IL-6 and VICs calcification were increased. However, after treated with LPAR antagonist, the increase of IL-6 and the calcification of VICs induced by LPCs could be prevented (Bouchareb et al., 2015), indicating that LysoPA, which was transformed by ATX into a final product, had stronger pro-inflammatory and calcification activities, consolidating the conjecture that ATX and its metabolites LPC and LysoPA could mediate AV calcification through the NF-κB—IL-6—BMP pathway.

In addition, both Lp(a) and OxPL can directly stimulate the NF-κB signaling pathway through MAPK. Yu et al., (2017) observed that increased expression of MAPK38 and MKK3/6 following an incubation with Lp(a) for 48 h in HAVICs isolated from non-calcified valves. At the same time, the matrix proteins and minerals secreted by HAVICs such as ALP, phosphate, BMP-2 and BMP-4 were also increased. While the expression of these matrix proteins and minerals were decreased after the use of MAPK38 inhibitors. (Yu et al., 2017). Since MAPK is induced by p38 and the NF-κ B signaling pathway, these findings indicate that Lp(a) and OxPL can also stimulate the p38/NF-κB—BMP signaling pathway to induce AV calcification by increasing MAPK expression. Therefore, Lp(a) and OxPL can elevate the conversion of LPC to LysoPA by increasing the activity of ATX, and subsequently stimulate both the NF-κB—IL-6—BMP pathway and MAPK—p38—NF-κB—BMP pathways to promote AV calcification.

#### 4.2.4 OxPL Promotes Inflammation in CAVD

The process involved in fiber calcification of AV remodeling mediated by inflammation is highly complx (Raddatz et al., 2019). In surgically resected calcified aortic valves, it was found that in addition to the normal valve tissue, inflammatory immune cells including macrophages, T cells and B cells (Raddatz et al., 2019) and various inflammatory mediators were also present (Winchester et al., 2011; Heistad et al., 2013), indicating the existence of the immune activation in the development of CAVD.

Lp(a)-mediated a pro-inflammatory response through OxPL (van der Valk et al., 2016), which can promote the inflammation of ECs by increasing the expression of pro-inflammatory cytokines (e.g., IL-1) (Subbanagounder et al., 2000). A study found that patients with an IL-1 (+) genotype were associated with an increased risk of developing CAVD, whereas patients with an IL-1 (-) genotype were not sensitive to the increased incidence of CAVD mediated by OxPL/apoB or Lp(a) (Tsimikas et al., 2014). This finding was also confirmed in subjects with elevated hsCRP levels (Tsimikas et al., 2014). It is suggested that there is a clinical biological correlation between genetic susceptibility to IL-1, OxPL, Lp(a), and genetic susceptibility to CAVD events, and that this biological relationship is mediated by the inflammatory response.

It is worth noting that in the process of CAVD, the pro-inflammatory effect of Lp(a) is primarily mediated by OxPL, not only by directly inducing the expression of related inflammatory genes (Serbulea et al., 2018), but also manifested through bone-forming signals (for example, OxPL can indirectly activate IL-6 through the ATX-mediated transduction of NF-κB signaling to promote an AV inflammatory response (Bochkov et al., 2010)). Furthermore, it can also change the gene expression in valvular interstitial cells to thicken and calcify the valve via the above-defined mechanism.

The above studies suggest that Lp(a) and OxPL may promote CAVD by directly inducing the expression of related inflammatory genes, IL-1, and the NF-κB—IL-6 pathway. This pro-inflammatory effect may play an important role in the early stage of valvular sclerosis.

#### 4.2.5 OxPL Promotes Oxidative Stress in CAVD

It has been confirmed that oxidative stress increases in AVS valve and is related to the decoupling pathway of nitric oxide synthase (NOS) (Miller et al., 2008). In addition, NADPH oxidase expression is increased in surgically excised calcified AV, and this process contributs to the production of reactive oxygen species (Liberman et al., 2008). These findings suggested that oxidative stress is also an important factor involved in CAVD.

Studies have shown that the role of Lp(a) in promoting calcification may be produced through the oxidative stress pathway induced by OxPL. Mody et al., (2001), Liberman et al., (2008) found that the production of reactive oxygen species (ROS) around calcified deposits was increased in human calcified AV, as well as the levels of OxPL and NAD(P)H oxidase subunit, Nox2. Furthermore, the production of ROS and OxPL levels were more significantly increased after the use of antioxidants, suggesting that OxPL-mediated ROS production near the calcified region can accelerate the CAVD progression. *In vitro*, results showed that an increase in reactive oxygen species, such as the bicyclic endoperoxides IsoP-PC (or G2-IsoP-PC) and F2-IsoP-PC produced by the oxidation of PAPC (Lu et al., 2017), can promote the AV calcification (Tsimikas and Witztum, 2008). An increased level of F2-IsoP-PC was considered to be the best indicator of endogenous oxidative stress (Liu et al., 2009; Milne et al., 2011). It is suggested that OxPL may mediate oxidative stress-induced AV calcification through the production of F2-IsoPs. However, while there are still few studies describeing Lp(a)- and OxPL-mediated CAVD through oxidative stress, the specific mechanism remains to be further elucidated.

### 4.3 Activating the Wnt Pathway Directly

The Wnt signal consists of 19 wingless lipid modified glycoproteins in mammals (Rao and Kühl, 2010). When some phosphoprotein intracellular is activated, it modulates 3 downstream signaling pathways:1. canonical (β-catenin-dependent) Wnt pathway; 2. non-canonical planar cell polarity (PCP) pathway; 3. non-canonical Wnt/Ca+2 pathway (Komiya and Habas, 2008). Wnt signaling plays a vital role in determining cell fate, spindle formation, polarity, organogenesis, angiogenesis and aortic valve calcification (Yamaguchi, 2001; Logan and Nusse, 2004).

The canonical Wnt pathway plays an important role in aortic valve calcification. First, BMP-2 can be detected in valvular interstitial cells isolated from the calcified aortic valves of aged rats, and has been shown to increase the ALP expression through the Wnt/β-catenin signaling pathway and stimulate calcification and cartilage formation. Second, MSK-2 has also been confirmed to cause aortic valve calcification through the Wnt/β-catenin signaling pathway. Third, the Wnt/β-catenin pathway is crucial for the induction of RUNX2. Both Wnt protein and T-cell factor-1 can directly bind to the proximal promoter of RUNX2. Once RUNX2 is induced to express, these cells will enter a state of osteoblast differentiation and up-regulated the expression of bone-related proteins (e.g., osterix, osteocalcim, and sclerostin) (Yu et al., 2017).

Yu et al., (2017) found that after co-incubation of HAVICs with Lp(a) for 48 h, the phosphorylation of glycogen synthase 3α/β (GSK3α/β) and the nuclear translocation of β-catenin was increased. What’s more, the expression of matrix proteins and minerals (e.g., ALP, phosphate, and osterix) and cytokines (OSF-2, RUNX-2, MSK-2, Wnt3 α, and BMP-2) secreted by HAVICs increased at the same time. Expression of ALP, BMP-2, and calcification were further increased after a co-incubation for 3 weeks. However, after added GSK3α/β inhibitors, they found that GSK3β inactivation, β-catenin degradation, ALP activity, and calcium deposition were significantly decreased. It can be confirmed that Lp(a) can simultaneously induce the expression of GSK3β in the canonical Wnt signal pathway through BMP and MSK-2 to increase the expression of β-catenin, RUNX2, ALP, and other ossification-related proteins to drive HAVICs calcification.

In addition, non-canonical Wnt pathway (such as non-canonical Wnt/Ca+2 pathway, PCP pathway.) also play an important role in CAVD. It has been confirmed that the levels of Wnt5 and Wnt11 in calcified aortic valves were higher than those in normal valves, especially Wnt5b. Study found that Wnt5b and Wnt11 were also increased when co-incubated with Lp(a), while calcification induced by Lp(a) was decreased in HAVICs after the use of a GSK3 α/β inhibitor (Yu et al., 2017). This finding confirmed that Lp(a) could also drive the occurrence and development of aortic valve calcification through the Wnt5b-induced Wnt/Ca2+pathway and Wnt11-induced PCP pathway.

### 4.4 Lp-PLA_2_ Is Another Pathogenic Factor of Lp(a) Causing CAVD

Lipoprotein-associated phospholipase A2 (Lp-PLA2) is secreted and released into the blood by macrophages and other inflammatory cells. Moreover, Lp-PLA2 can be combined and transported with apoB-rich lipoprotein particles, such as Lp(a), LDL, and HDL (Tellis and Tselepis, 2009). Among these, Lp(a) transports Ox-PL in the blood through the top of Lp-PLA2. Studies have confirmed that there is an independent positive correlation between circulating Lp-PLA2 levels and calcified aortic valve stenosis (Capoulade et al., 2015b). A number of prospective observational studies have shown that the quality and/or activity of circulating Lp-PLA2 are related to the risk of CAVD (Ballantyne et al., 2004; Tsimikas et al., 2009). From the above studies, it is speculated that Lp(a) may cause CAVD through Lp-PLA2.

It has been confirmed that the expression of Lp-PLA2 is increased in the stenotic aortic valve (Kolasa-Trela et al., 2012; Mahmut et al., 2014a, Mahmut et al., 2014b), and is associated with the valve fibrous calcification remodeling (Mahmut et al., 2014a, Mahmut et al., 2014b). A small study demonstrated that patients with calcific aortic valve stenosis (CAVS) had higher Lp-PLA2 levels compared with the control group. Kolasa-Trela et al., (2012) Similarly Perrot et al., (2020) detected Lp-PLA2 activity in 890 patients undergoing cardiac surgery and found that Lp-PLA2 activity was positively correlated with the presence of CAVS. Other studies performed by this team have shown that higher Lp-PLA2 activity was related to the progress of CAVS and the structural degradation of bioprosthesis (Mahmut et al., 2014b). Investigations into the studies related mechanism have shown that Lp-PLA2 can convert OxPL into LysoPc, which can lead to the loss of mitochondrial membrane potential and VICs apoptosis, followed by increasing the deposition of hydroxyapatite in aortic valve to promote VICs calcification (Lehti et al., 2013; Mahmut et al., 2014a; Bouchareb et al., 2015), ultimately resulting in CAVD.

However, Perrot et al., (2020) simultaneously selected four SNP loci (rs7756935, rs1421368, rs1805017, and rs4498351) related to Lp-PLA2 quality or activity in PLA2G7, and conducted a genetic association analysis of eight cohort studies (a total of 10,137 CAVS cases and 434,585 controls). Meta-analysis revealed that these four SNP were not associated with CAVS, suggesting that Lp-PLA2 is unlikely to represent a causal risk factor or therapeutic target of CAVS.

There are currently few published studies on Lp(a)-Lp-PLA2, and it is speculated that its function may be similar to that of LDL - Lp-PLA2. Unfortunately, the results of relevant clinical studies are inconsistent, and there are fewer studies related to the mechanism between Lp-PLA2 and CAVD. Therefore, further research is required to support the relationship between the Lp-PLA2 and CAVD and the associated mechanism.

### 4.5 Other Mechanisms

Lp(a) may also promote inflammation through apoB due to the single molecular structure of apoB_100_. The ApoB-derived peptide, ApoBDS-1, can promote the production of pro-inflammatory cytokines (e.g., IL-8) and other pro-inflammatory mediators (e.g., prostaglandin E2) (Wiesner et al., 2013). It has been confirmed that the arachidonic acid (AA) pathway, which produces prostaglandin E2, plays an important role in aortic valve calcification (Nagy et al., 2011). It has also been found that the expression of 5-oxyesterase is required for increased leukotriene synthesis in calcified AV, and leukotriene C4 can promote both BMP-2 and BMP-6 expression of and VICs calcification (Nagy et al., 2011). Moreover, increased expression of cyclooxygenase 2 (COX2) has been observed in VICs isolated from narrow AVs. Wirrig et al., (2015) confirmed that aortic valve calcification was decreased in Klotho-deficient mice that lack COX2 function, however, there are currently few related studies.

In addition, ApoC-III may be involved in Lp(a)-induced CAVD. Capoulade et al., (2020) found that the ApoC-III immunostaining was positive in patients with pathological grade 1–4 aortic lobules. Furthermore, the ApoCIII-Lp(a) complex has been found to exist in all aortic valve stenosis patients, and the risk of AVR associated with cardiac death was significantly increased in patients in the highest quartile for apoCIII-Lp(a) and Lp(a) (Capoulade et al., 2020). Therefore, the ApoCIII-Lp(a) complex may be related to calcified aortic valve stenosis, but the specific mechanism must be further explored.

## 5 Current Status and Treatment Prospect

Observational studies have found that Lp(a) is an independent risk factor for CAVD. Genomic studies have also shown that there is a causal relationship between Lp(a)-related gene polymorphism and aortic valve calcification.

Only 2 trials are listed in clinicaltrials.gov evaluating medical therapy for Lp(a)-mediated CAVD (insert Table 3 here). These include EAVaLL, describing the use niacin versus placebo, and a study of PCSK9 inhibitor, which is in phase II, but both study is recruiting very slowly.

**TABLE 3:** Trials of medical therapy for treatment of Lp(a)-mediated CAVD.

Study	NCT No.	Intervention	Phase/Status	No. of patients	Main Inclusion Criteria	Main primary endpoint	Main secondary endpoint
EAVaLL	NCT02109614	Niacin 1,500–2000 mg vs Placebo 1500 mg	Early I/Unknown	238	Age >50 years and <85 years, aortic sclerosis OR mild AS, Lp(a) > 50 mg/dl (>80th percentile)	Calcium score progression by cardiac CT	Mean change in Lp(a) levels between treatment arms, Change in peak velocity (in m/s); Change in mean gradient (in mmHg); Change in AV area (in cm2)
PCSK9 Inhibitors in the Progression of Aortic Stenosis	NCT03051360	PCSK9 Inhibitor vs Placebo	II/Unknown	140	Diagnosis of aortic stenosis (mild to moderate), LDL-C > 70 mg/dl at baseline	Progression of the Calcium score measured by cardiac CT (Agatston score) and by NaF PET	Efficacy of inhibition in calcium score progression (Agatston score) by the presence of Lp(a) SNPs.

EAVaLL, Early Aortic Valve Lipoprotein(a) Lowering Trial.

There is currently a lack of evidence from randomized controlled trials (RCT) to show that lowering Lp(a) levels can improve CAVD and cardiovascular outcomes. Therefore, more RCT that reduce Lp(a) levels without affecting other lipid and non-lipid risk factors are important ways to directly address the hypothesis are needed.

**FIGURE 3:**
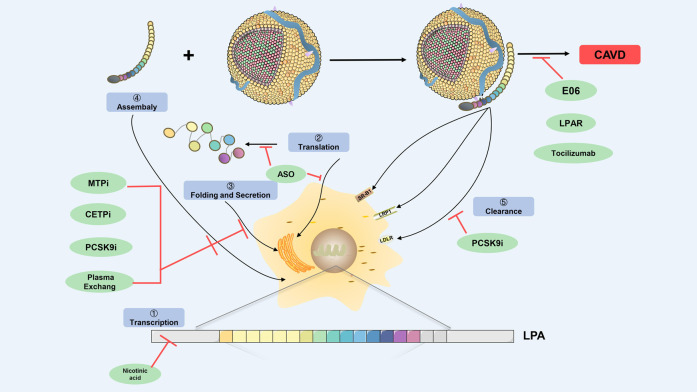
Prospecting treatment of Lp(a)-mediated CAVD. Schematic diagram depicts regulation points for Lp(a) biosynthesis and catabolism (highlighted in blue boxes) as a prospecting treatment target. The green ovals indicate therapeutic agents that have been shown to modulate Lp(a) levels, and potentially Lp(a) pathogenic affects. The prospecting treatment target at which they act is shown with red lines. CETPi, CETP inhibitor; PCSK9i, PCSK9 inhibitor; ASO, antisense oligonucleotides; MTPi, microsome triglyceride transfer protein inhibitor; LRP-1, low-density lipoprotein receptor-related protein-1; SR-B1, scavenger receptor B1; LDLR, low-density lipoprotein receptor; CAVD, calcific aortic valve disease.

However, the process of Lp(a)-induced CAVD is mainly mediated through OxPL, ATX, Lp-PLA2, and apoB, which are involved in aortic valve calcification and inflammation. Since statins did not delay the progression of CAVD, whether the relevant new Lp(a)-lowering drugs drugs can effectively prevent and delay the progression of CAVD is of great significance. (Insert Figure 3 here).

### 5.1 Antisense Oligonucleotides

The heterogeneity of apo(a) in Lp(a) determines the change of Lp(a) plasma levels, suggesting that reducing apo(a) can reduce the Lp(a).

RCT have shown that the antisense nucleotides IONIS-APO(a)-Rx and IONIS-APO(a)-LRx of apo(a) mRNA could reduce Lp(a) levels (Viney et al., 2016). IONIS-APO(a)-Rx resulted in a dose-dependent reduction of 66–92% Lp(a) level and 45% OxPL-apo(B) and 25% OxPL-apo (a) levels respectively after 36 days treatment in patients with high Lp(a) levels (Hutcheson et al., 2016). In 3 randomized, double-blind, placebo-controlled trials, it was found that Lp (a) levels decreased by 66, 80, and 92% in IONIS-APO (a)-LR 10, 20 and 40 mg/weeks, respectively, suggesting that IONIS-APO(a)-LR decreased Lp(a) significantly in a dose-dependent manner (Viney et al., 2016).

Recent, a randomized, double-blind, placebo study of 286 patients with confirmed cardiovascular disease from 5 countries treated with hepatocyte-directed antisense oligonucleotide AKCEA-APO(a)-LRx (TQJ230) for 6–12 months, the results showed an average decrease of 35% in 20 mg group, 56% in 40 mg group, 58% in 20 mg group, 72% in 60 mg group every 4 weeks, and 80% in 20 mg group weekly, while the placebo group only decreased 6% (Tsimikas et al., 2020), further confirming that AKCEA-APO(a)-LRx treatment can decreased Lp (a) levels in a dose-dependent manner with good safety.

These drastically lower Lp(a) data indicate that these drugs are expected to attenuate the process of CAVD. Additional studies are required to confirm the prevalence of lowering Lp(a) levels can improve CAVD and cardiovascular outcomes.

### 5.2 PCSK9 Inhibitors

A loss-of-function mutation in proprotein converting enzyme subtilisin/kexin type 9 (PCSK9) gene can lead to a significant decrease in LDL-C levels. PCSK9 inhibitors can not only reduce the level of LDL-C, but also effectively reduce the level of Lp(a). Studies have shown that the monoclonal antibody inhibitors, alirocumab and evolocumab, can reduce LDL-C while also reducing plasma Lp(a) levels by up to 30% (Hovingh et al., 2015).

*In vivo*, studies have shown that PCSK9 can regulate the level of Lp(a) by promoting the internalization of LDL-R (Hovingh et al., 2015). In addition, metabolic tracer studies have shown that PCSK9 inhibitors can inhibit the synthesis of Lp(a) (Sabatine et al., 2015). The subgroups analysis results of the FOURIER (Bergmark et al., 2020) and the GLAGOV studies (Nicholls et al., 2016) have shown that evolocumab can provide additional benefits for reducing the risk of aortic valve stenosis in patients with ASCVD, which not only adds to the evidence that decrease Lp(a) levels are related to the occurrence of AVS, but also lays a solid foundation for PCSK9 inhibitors to occupy a place in the treatment of AVS.

### 5.3 Mechanism-Related Drugs

Since OxPL plays a key role in CAVD, targeting or neutralizing OxPL may represent a therapeutic option. *In vitro*, mice with an *Ldlr* ^*-/-*^ background expressing E06 single chain variable fragment (*Ldlr* ^−/−^/E06-scFc could neutralizing OxPL) showed a reduction in the AV pressure gradient by 49%, and decreased total calcium content compared with *Ldlr* ^*-/-*^ mice. What’s more, *Ldlr* ^*-/-*^ mice could not survive, whereas all *Ldlr* ^−/−^/mice treated with E06-scFc survived (Que et al., 2018), suggesting that E06 may alleviate the increased pressure gradient caused by aortic stenosis and improve the survival rate of *Ldlr* ^−/−^ mice. However, these findings remains limited to animal experiments, and further clinical research on E06 is needed.

Anti-inflammatory therapy may provide another treatment option for patients with CAVD. Tocilizumab, a monoclonal humanized mouse antibody inhibitor of IL-6 receptor, antagonizes IL-6 signaling by blocking the binding of IL-6 to its corresponding receptor, which has been approved for the treatment of rheumatoid arthritis (Karkhur et al., 2019). *In vitro*, studies have shown that neutralization of IL-6 with anti-IL-6 antibody can down-regulate the expression of BMP2 and RUNX2, thereby reducing the occurrence of vascular calcification (Callegari et al., 2014). Therefore, anti-IL-6 therapy is expected to become another target for CAVD patients with elevated Lp(a) levels.

In addition, LPAR antagonists become another promising drug for the prevention and treatment of CAVD because they can block the secretion of IL-6 induced by LPC. Studies have reported that targeting LPAR can reduce the calcification of VIC (Palmer et al., 2018). Consistent with *in vitro* experiments, *in vivo*, studies have shown that LPAR-deficient mice exhibit reduced osteoblast differentiation, which in turn leads to decreased in bone mass (Bouchareb et al., 2015). What’s more, in patients with idiopathic pulmonary fibrosis, LPAR antagonists can significantly reduce the rate of disease progression (Harpel et al., 1989). However, it should be noted that although most patients are well tolerant to LPAR antagonists, elevated liver transaminase and ALP are still detected in some patients (Harpel et al., 1989).

### 5.4 Other Drugs

Nicotinic acid (Insull et al., 2004; Tuteja et al., 2018), apoB-lowering drugs (e.g., lometazepide (Benjamin et al., 2017) and mipomesan (Thomas et al., 2013)), CETP inhibitors (e.g., anacetrapib) (Cannon et al., 2010; Äijänen et al., 2014) and plasma exchange (Leebmann et al., 2013; Waldmann and Parhofer, 2016; Klingel et al., 2017) can significantly reduce plasma Lp(a) levels. Although there continues to be lack of studies that are directly related to CAVD and a definite efficacy in reducing Lp(a), they may represent potential therapeutic targets for CAVD.

## 6 Summary

CAVD is extremely common, and there is no effective drug treatment available at present. It has been proved that while surgical valve replacement is effective for advanced disease, it is expensive, the treatment time is limited, and it is not the best choice for elderly patients. Therefore, there is an increased demand for the non-invasive treatment of CAVD patients.

Although lipid deposition and inflammation may play a role in the early stages of CAVD, the later stage of calcification and calcification is a continuous process characterized by calcium deposition and osteoblast formation. This means that once the disease enters the progressive stage, most drugs may be ineffective. Therefore, in the early stage of CAVD, that is, before the occurrence of valvular stiffness and blood flow obstruction, it may be the best optimal time for related drugs to exert their potential therapeutic effects. High Lp(a) is an independent risk factor for CAVD, and close attention should be paid to the level of Lp(a) in this disease risk assessment. However, there is currently a lack of trials using a reduction in Lp(a) as a primary endpoint in CAVD, and many therapies that can significantly reduce Lp(a) remain clinical trials. We look forward to further exploration of the pathogenic mechanism of Lp(a) as well as the development of active and effective means for CAVD prevention and treatment. We also anticipate that this field will move quickly from the bench to the bedside.
